# Initial Results of the Enhanced Recovery After Surgery (ERAS) Program in Patients Undergoing Lobectomy in the Treatment of Lung Cancer: An Experience From the University Medical Center Ho Chi Minh City

**DOI:** 10.7759/cureus.57870

**Published:** 2024-04-08

**Authors:** Ho Tat Bang, Tran Thanh Vy, Nguyen Van Tap

**Affiliations:** 1 Department of Thoracic and Vascular, University Medical Center Ho Chi Minh City, Ho Chi Minh City, VNM; 2 Department of Health Organization and Management, University Medical Center Ho Chi Minh City, Ho Chi Minh City, VNM; 3 Department of Medical Management, Nguyen Tat Thanh University, Ho Chi Minh City, VNM

**Keywords:** length of hospital stay (los), postoperative recovery, lung cancer, lobectomy, enhanced recovery after surgery (eras)

## Abstract

Background: Lobectomy is a standard surgical method in the treatment of early stages of non-small cell lung cancer (NSCLC). The enhanced recovery after surgery (ERAS) program aims to reduce the postoperative length of hospital stay (PLOS) in major surgeries. This study evaluated the impact of the ERAS program on PLOS and identified related factors in patients undergoing lobectomy for NSCLC.

Methods: This prospective observational study was conducted at the University Medical Center Ho Chi Minh City, Vietnam, from February 2022 to December 2023. We included patients diagnosed with NSCLC scheduled for lobectomy. The ERAS protocol was applied according to guidelines from the ERAS Society and the French Society of Anaesthesia and Intensive Care Medicine. We collected data on patient demographics, surgical details, adherence to the ERAS protocol, and postoperative outcomes, including the PLOS.

Results: Among the 98 patients enrolled, the median PLOS after ERAS intervention was 4.1 days (interquartile range: 3.7 to 5.2 days). Adherence to ERAS protocols significantly correlated with reduced PLOS (p<0.001). Notably, smoking status was identified as a related factor of PLOS (p=0.002). Complications (p<0.001), surgical method (p=0.007), operation time (p<0.001), duration of postanesthesia care unit (p=0.006), duration of thoracic drainage (p<0.001), and urinary catheter retention time (p=0.023) were also associated with PLOS variations.

Conclusion: Implementing the ERAS program in patients undergoing lobectomy for NSCLC at our center reduced PLOS and highlighted the importance of protocol adherence for optimizing surgical outcomes. These findings supported the broader adoption of ERAS protocols in thoracic surgery to enhance patient recovery. Future research should focus on multi-center studies to generalize these results and further dissect the impact of individual ERAS components.

## Introduction

Lobectomy remains the widely recognized method for treating non-small cell lung cancer (NSCLC) in its early stages despite the significant risks that accompany such major surgical interventions [1,2]. The recovery process after surgery can be slow and complicated due to perioperative complications, as well as issues in care and treatment that can result in prolonged hospital stays [3]. Our center's research has shown that factors contribute to these prolonged stays, including factors related to the patients themselves and issues in care [4].

The enhanced recovery after surgery (ERAS) program has proved to be a crucial step forward in the care and treatment of surgical patients, reducing hospital stays and improving postoperative clinical outcomes in patients undergoing major surgeries [5-7]. Studies on the effectiveness of the ERAS model in gastrointestinal surgeries are very common, but thoracic and lung surgeries are limited [8]. The ERAS Society has updated its guidelines for implementing ERAS for lung surgery patients since 2019 [9]. Studies have also demonstrated the role of ERAS applications in minimizing complications and the length of hospital stays, limiting medical costs, and reducing hospital overload in lung surgery patients [10,11]. The efficacy of the ERAS program for lung cancer patients undergoing lobectomy showed a reduction in the median hospital stay by 1-3 days compared to the group not participating in the ERAS program [10]. ERAS can benefit elderly lung cancer patients undergoing surgery by reducing hospital stay, improving pulmonary function, reducing inflammation, and lowering the risk of complications [12]. However, the application process varies depending on the population characteristics, facilities, and experience of each medical facility and the characteristics of each country's health system.

Research at University Medical Center Ho Chi Minh City (UMC HCMC) prior to the ERAS intervention revealed that the median length of postoperative hospital stay after lobectomy in the treatment of lung cancer was 5.2 days, with over half of the patients staying for more than five days. Many issues were identified during the care and treatment process that contributed to the extended hospital stay [4]. In 2022, the UMC HCMC has begun applying the ERAS program for patients undergoing lobectomy surgery to treat lung cancer. A study was conducted to report the initial results of the ERAS program in patients undergoing lobectomy in lung cancer treatment.

## Materials and methods

Objectives

The general objective is to investigate the postoperative length of hospital stay (PLOS) and related factors in a group of patients diagnosed with NSCLC who underwent lobectomy and participated in the ERAS program.

The specific objectives include (1) investigating the relationship between adherence with ERAS procedures and postoperative hospital stay after lung lobectomy for cancer treatment and (2) determining the relationship between the PLOS and the pathological characteristics, characteristics of the treatment, and care process in patients who participate in the ERAS program after lobectomy to treat lung cancer.

Study settings and participants

This prospective observational study was conducted at the UMC HCMC, Ho Chi Minh City, Vietnam, from February 2022 to December 2023. The Ethics Council in Biomedical Research of the University of Medicine and Pharmacy in Ho Chi Minh City issued approval (2294/ĐHYD). The study included patients diagnosed with NSCLC through biopsy who were scheduled to undergo a lobectomy and receive care in accordance with the hospital's ERAS program. Additionally, patients with a diagnosed lung tumor, but without yet having pathology results, and scheduled for a lobectomy were also cared for according to the ERAS protocol. Patients were excluded from the study if their postoperative pathology results did not indicate cancer. The study further excluded patients who underwent surgery at UMC HCMC but were transferred to another hospital for postoperative care. An informed consent form was obtained from all patients prior to enrollment, and they were operated on and cared for by the same surgical team.

ERAS pathway at UMC

**Figure 1:**
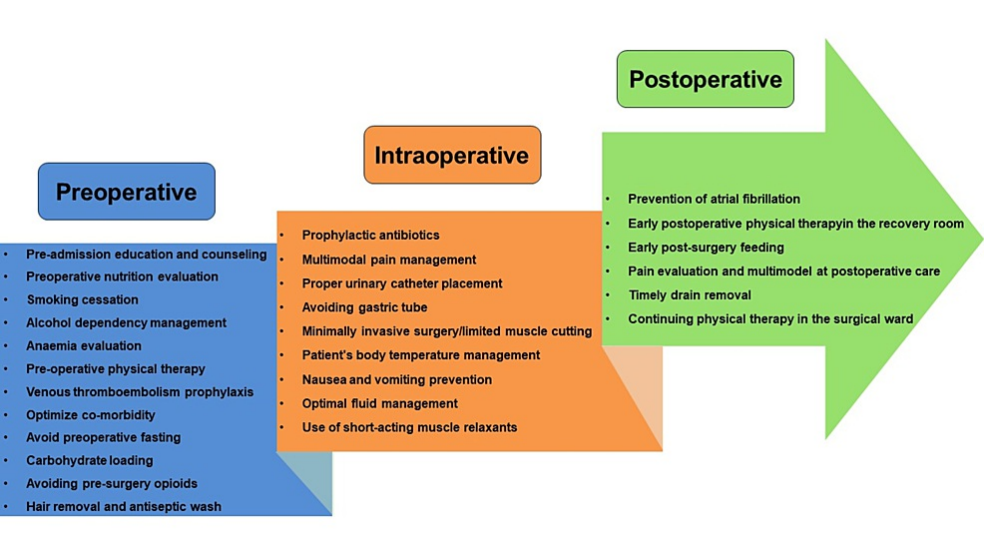
The multi-disciplinary coordination process with many activities before, during, and after surgery, according to the ERAS program ERAS: Enhanced recovery after surgery

The procedure for operating the ERAS program at the UMC HCMC was designed based on the ERAS Society guidelines [9] and guidelines on enhanced recovery after pulmonary lobectomy from the French Society of Anaesthesia and Intensive Care Medicine [13]. The process of developing the ERAS process was carried out after assessing the current situation of patient care after lung cancer surgery, the current state of facilities, and surveying the knowledge, attitudes, and practices of medical staff as well as consulting with the hospital Scientific Council. The multi-disciplinary coordination process with many activities before, during, and after surgery was as follows (Figure 1).

The preoperative phase included 12 factors: (1) preadmission education and counseling; (2) preoperative nutrition evaluation; (3) smoking cessation; (4) alcohol dependency management; (5) anemia evaluation; (6) preoperative physical therapy; (7) venous thromboembolism prophylaxis; (8) optimize comorbidity; (9) avoid preoperative fasting; (10) carbohydrate loading; (11) avoiding presurgery opioids; and (12) hair removal and antiseptic wash.

The intraoperative phase included nine factors:(1) prophylactic antibiotics; (2) multimodal pain management; (3) correct urinary catheter placement; (4) avoiding gastric tube; (5) minimally invasive surgery/limited muscle cutting; (6) patient's body temperature management; (7) nausea and vomiting prevention; (8) optimal fluid management; and (9) use of short-acting muscle relaxants.

The postoperative phase included six factors:(1) prevention of atrial fibrillation; (2) early postoperative at recovery room; (3) early postsurgery feeding; and (4) pain evaluation and multimodel at postoperative care; (5) timely drain removal; (6) continuing physiotherapy in the surgical ward.

Data collection and tools

Collected research variables included general characteristics (gender, body mass index (BMI), place of living); characteristics of risk factors and comorbidities (smoking, comorbidities); pathological characteristics of lung cancer (disease detection situation, symptoms at onset, symptoms, time of symptom detection, tumor location, TNM stage, cancer cell type, degree of differentiation); characteristics of the treatment and care process (including surgical approach, complications, amount of blood loss, surgery time, time in the recovery unit, time of pleural tube placement, time of postoperative fasting time, urine catheterization time). Evaluation of adherence to the ERAS process steps was performed by the ERAS supervisor team. The ERAS supervisor team had been fully trained and unified in the supervision process. The results of monitoring would be re-evaluated by the head of the monitoring group. The tool was an electronic checklist attached directly to the medical record. All collected information is entered into a precompiled data collection form.

Study outcome

The study's endpoint was the PLOS, which was determined by calculating the duration (hours) from when the patient left the operating room to the time of hospital discharge. This difference was converted into day units and rounded to one decimal place. The hospital discharge criteria are as follows: (1) pleural drainage tube removed; (2) no evidence of infection; (3) pain control with oral analgesics; (4) X-ray reveals good lung expansion, no major effusion or air effusion; and (5) ability to feed orally and did not require nasal cannula oxygen. In addition, the factors related to the length of hospital stay after surgery would be examined, along with baseline characteristics, pathological characteristics, and adherence to the ERAS protocol.

Statistical analysis

The study data were analyzed with Stata software (version 16.0, Stata Statistical Software: Release 16, StataCorp LLC, College Station, United States). The two-sample Wilcoxon rank-sum and Kruskal-Wallis rank test were employed to compare the median of PLOS between groups when PLOS had an irregular distribution. Spearman correlation was used to evaluate the correlation between quantitative variables with non-normal distribution. Statistical tests are significant when p-value <0.05.

## Results

The study enrolled 98 lung cancer patients who underwent lobectomy surgery and participated in the ERAS program. Patient characteristics are presented in Table 1. Of these, 55 (56.1%) patients were male, and 58 (59.2%) were aged 60 or above. A significant number of participants, 33(33.7%), resided in urban areas. Regarding BMI, 60 (61.2%) of the patients had a normal BMI, 3 (3.1%) exhibited malnutrition, and 35 (35.7%) were overweight or obese. According to the American Society of Anesthesiologists classification, 49 (50%) patients were classified as level II, followed by 43 (43.9%) as level III. Non-smokers comprised 70 (71.4%) of the study population, with current smokers at 10 (10.2%) and former smokers at 18 (18.4%). The most frequently observed comorbidities were hypertension with 51 (52%), diabetes mellitus with 13 (13.3%), and coronary artery disease with 11 (11.2%) patients.

**Table 1:** Characteristics of patients (n=98) COPD: Chronic obstructive pulmonary disease; TNM: Tumor-nodes-metastases; ASA: American Society of Anesthesiologists; BMI: Body mass index

Variables		Frequency (N)	Proportion (%)
Gender	Male	55	56.1
	Female	43	43.9
Age group	<60	40	40.8
	>=60	58	59.2
Living place	Urban area	33	33.7
	Rural area	65	66.3
BMI classification (BMI = weight in kilograms (kg) divided by the square of height in meters (m^2^))	Underweight (BMI<18.5)	3	3.1
	Normal (BMI 18.5-22.9)	60	61.2
	Overweight (BMI≥23)	35	35.7
ASA classification	I	6	6.1
	II	49	50
	III	43	43.9
Cigarette smoking status	Never	70	71.4
	Current	10	10.2
	Former	18	18.4
Comorbidities	Hypertension	51	52.0
	Asthma/COPD	4	4.1
	Diabetes	13	13.3
	Dyslipidemia	1	1.0
	Coronary artery disease	11	11.2
	Heart failure	2	2.0
	Sequelae of stroke	2	2.0
	Thyroid dysfunction	6	6.1
	Anemia	1	1.0
	Other cancers	2	2.0
	Other diseases	9	9.2
Symptom onset	Symptomatic	51	52.0
	Asymptomatic	47	48.0
Symptoms (n=51)	Persistent cough	23	45.1
	Chest pain	12	23.5
	Weight loss	2	3.9
	Loss of appetite, fatigue	12	23.5
	Hemoptisi	2	4.0
Duration of illness (n=51)	Less than one month	18	35.3
	From one month to less than three months	11	21.6
	From three months to less than six months	12	23.5
	From six months or more	10	19.6
Tumor location	Left-upper lobe	21	21.4
	Left-lower lobe	21	21.4
	Right-upper lobe	21	21.4
	Right-lower lobe	22	22.5
	Right-middle lobe	13	13.3
TNM stage	IA	22	22.5
	IB	13	13.3
	IIA	24	24.5
	IIB	20	20.4
	IIIA	16	16.3
	IIIB	3	3.1
Lung cancer cell types	Adenocarcinoma	81	82.7
	Squamous cell carcinoma	17	17.3
	Large cell carcinoma	0	0.0
Cell differentiation	Well-differentiated	10	10.2
	Moderately differentiated	77	78.6
	Poorly differentiated	11	11.2

Symptoms were observed in 51 (52%) patients, with the most common being prolonged cough in 23 (45.1%), chest pain, and fatigue or appetite loss in 12 (23.5%). Tumor distribution across the lung lobes was relatively even, with the right middle lobe being the least affected in 13 (13.3%) patients. The TNM classification showed that stage IIA was present in 24 (24.5%) and IIB in 20 (20.4%) cases. Adenocarcinoma emerged as the dominant histological type, accounting for 81 (82.7%) cases, with the majority of tumors classified as moderately differentiated, 77 (78.6%).

The median PLOS after ERAS intervention was 4.1 days (interquartile range from 3.7 to 5.2 days). The total median length of hospital stay was reported to be 7.3 days (interquartile range from 6.9 to 10.1 days). Table 2 describes the association between PLOS and baseline characteristics. The results showed that the median PLOS in the smoking group was 6.9 days, 2.5 days higher than the non-smoking group, and 3.2 days higher than the group that smoked but had quit (p=0.002). There was no relationship between PLOS and other factors.

**Table 2:** Association between postoperative length of hospital stay and baseline characteristics (n=98) #: Two-sample Wilcoxon rank-sum test ##: Kruskal-Wallis rank test COPD: Chronic obstructive pulmonary disease; TNM: Tumor-nodes-metastases; ASA: American Society of Anesthesiologists; BMI: Body mass index

Variables		Postoperative length of hospital stay (day)		p-value
		Median	Interquartile range	
Gender	Male	4.2	3.7-5.0	0.545^#^
	Female	4.1	3.6-6.3	
Age group	<60	4.0	3.7-4.9	0.138^#^
	>=60	4.4	3.7-5.9	
Living place	Urban area	4.0	3.8-5.1	0.570^#^
	Rural area	4.6	3.8-5.8	
BMI classification (BMI = weight in kilograms (kg) divided by the square of height in meters (m^2^))	Underweight (BMI<18.5)	4.1	4.0-9.9	0.594^##^
	Normal (BMI 18.5-22.9)	4.1	3.7-5.1	
	Overweight (BMI≥23)	4.2	3.6-5.9	
ASA classification	I	5.6	4.9-6.8	0.075^##^
	II	4.0	3.6-5.0	
	III	4.2	3.9-5.7	
Smoking	Never	4.1	3.8-5.0	0.002^##^
	Current	6.9	5.2-8.2	
	Former	3.7	3.0-4.9	
Comorbidities	Hypertension	4.2	3.8-5.9	0.380^#^
	Asthma/COPD	4.1	3.1-5.0	0.590^#^
	Diabetes	4.1	3.8-4.2	0.745^#^
	Dyslipidemia	8.2	-	0.133^#^
	Coronary artery disease	4.1	3.6-5.9	0.605^#^
	Heart failure	3.7	3.6-3.8	0.258^#^
	Sequelae of stroke	5.0	4.9-5.0	0.366^#^
	Thyroid dysfunction	4.5	2.8-5.5	0.744^#^
	Anemia	7.8	-	0.163^#^
	Other cancers	3.7	2.9-4.6	0.366^#^
	Other diseases	4.7	4.2-5.2	0.301^#^
Symptom onset	Symptomatic	4.2	3.8-5.0	0.725^#^
	Asymptomatic	4.0	3.5-6.1	
Symptoms (n=51)	Persistent cough	3.8	3.0-5.5	0.190^##^
	Chest pain	4.0	2.9-6.0	
	Weight loss	6.4	5.0-7.8	
	Loss of appetite. fatigue	4.9	3.9-6.9	
	Hemoptisi	5.9	5.9-5.9	
Duration of illness (n=51)	Less than one month	4.9	3.9-5.9	0.131^##^
	From one month to less than three months	3.5	2.9-5.0	
	From three months to less than six months	4.9	3.4-8.9	
	From six months or more	4.0	3.0-6.3	
Tumor location	Left-upper lobe	3.8	3.6-6.3	0.750^##^
	Left-lower lobe	4.8	3.8-5.9	
	Right-upper lobe	4.0	3.8-4.9	
	Right-lower lobe	4.4	3-5.2	
	Right-middle lobe	4.8	4.0-5.2	
TNM stage	IA	4.0	3.5-5.0	0.573^##^
	IB	4.2	4.0-5.9	
	IIA	4.0	3.4-5.0	
	IIB	4.4	3.9-6.6	
	IIIA	5.1	3.8-6.8	
	IIIB	4.0	3.9-5.0	
Lung cancer cell types	Adenocarcinoma	4.2	3.7-5.2	0.442^#^
	Squamous cell carcinoma	4.0	3.7-5.0	
Cell differentiation	Well-differentiated	5.1	4.0-5.9	0.624^##^
	Moderately differentiated	4.1	3.7-5.0	
	Poorly differentiated	4.6	2.9-7.0	

Table 3 illustrates the association between PLOS and treatment characteristics. Patients undergoing open surgery experienced a median PLOS of 5.4 days, which was marginally longer by 0.2 days compared to those whose procedure was converted from video-assisted thoracoscopy (VATS) to open surgery, and significantly longer by 1.4 days than patients undergoing purely VATS (p=0.007). Additionally, patients without complications had a median PLOS that was four days shorter than those with postoperative complications (p<0.001). There was a strong positive correlation between the duration of thoracic drainage and PLOS (p<0.001), moderate positive correlations were observed between operative time and PLOS (p<0.001), and a weak positive correlation was noted between the duration of stay in the postanesthesia care unit and urinary catheter retention time with PLOS (p<0.05).

**Table 3:** The relationship between postoperative hospital stay and treatment characteristics (n=98) #: Two-sample Wilcoxon rank-sum test ##: Kruskal-Wallis test a: Spearman regression VATS: Video-assisted thoracoscopy; R: Spearman correlation coefficient

Variables		Postoperative length of hospital stay (day)		p-value
		Median	Interquartile range	
Surgical method	Open surgery	5.4	4.0-6.6	0.007^#^^#^
	VATS	4.0	3.5-5.0	
	VATS convert to open surgery	5.2	4.0-7.1	
Complication	Yes	8.0	6.9-10.9	<0.001^#^
	No	4.0	3.6-5	
Amount of blood loss (ml)		R=0.181		0.075^a^
Operation time (minutes)		R=0.36		<0.001^a^
Duration of postanesthesia care unit (hours)		R=0.28		0.006^a^
Duration of thoracic drainage (days)		R=0.75		<0.001^a^
Preoperative fasting time (hours)		R=0.17		0.098^a^
Urinary catheter retention time (hours)		R=0.23		0.023^a^

Table 4 shows the relationship between adherence to ERAS protocols and the PLOS. The findings indicated significant differences in the median PLOS when comparing patients adhering to ERAS protocols to those who did not. Specifically, the group that received smoking cessation counseling had a median hospital stay that was 2.1 days shorter than the group without counseling (p=0.002). Patients who performed physical exercises before surgery had a stay that was 1.4 days shorter than those who did not exercise (p=0.005). Proper intake of preoperative carbohydrates and effective multimodal pain management were associated with a reduction of one day in the PLOS compared to those who did not follow these protocols (p=0.015 and p<0.001, respectively). Correct urinary catheterization practices resulted in a 1.7-day shorter stay (p<0.001), while minimal intervention techniques led to a 1.9-day reduction (p<0.001). Fluid management protocols were associated with a 2.9-day shorter stay (p=0.046), and atrial fibrillation prevention was linked to a 3.5-day reduction. Early postoperative physical therapy and timely removal of drains were associated with 2.7 and 3 days shorter stays, respectively (p=0.004 and p<0.001).

**Table 4:** The relationship between the length of postoperative hospital stay and each adherence to ERAS factors (n=98) #: Two-sample Wilcoxon rank-sum test ERAS: Enhanced recovery after surgery

ERAS factors	Compliance	Frequency (%)	Length of postoperative hospital stay (day)		p-value^#^
			Median	Interquartile range	
Preoperative phase					
Preadmission education and counseling	Yes	98 (100)	4.1	3.7-5.2	-
	No	0 (0)	-	-	
Preoperative nutrition evaluation	Yes	98 (100)	4.1	3.7-5.2	-
	No	0 (0)	-	-	
Smoking cessation	Yes	88 (89.8)	4	3.6-5.0	0.002^#^
	No	10 (10.2)	6.1	5.2-8.1	
Alcohol dependency management	Yes	97 (99)	4.1	3.7-5.2	0.764^#^
	No	1 (1)	4.8	4.8-4.8	
Anemia evaluation	Yes	98 (100)	4.1	3.7-5.2	-
	No	0 (0)	-	-	
Preoperative physical therapy	Yes	85 (86.7)	4.1	3.6-5.0	0.005^#^
	No	13 (13.3)	5.5	4.0-8.1	
Venous thromboembolism prophylaxis	Yes	98 (100)	4.1	3.7-5.2	-
	No	0 (0)	-	-	
Optimize comorbidity	Yes	98 (100)	4.1	3.7-5.2	-
	No	0 (0)	-	-	
Avoiding preoperative fasting	Yes	98 (100)	4.1	3.7-5.2	-
	No	0 (0)	-	-	
Carbohydrate loading	Yes	82 (83.7)	4	3.6-5.1	0.015^#^
	No	16 (16.3)	5	4.4-6.1	
Avoiding presurgery opioids	Yes	98 (100)	4.1	3.7-5.2	-
	No	0 (0)	-	-	
Hair removal and antiseptic wash	Yes	97 (99)	4.1	3.7-5.2	0.086^#^
	No	1 (1)	14.8	14.8-14.8	
Intra-operative phase					
Prophylactic antibiotics	Yes	98 (100)	4.1	3.7-5.2	-
	No	0 (0)	-	-	
Multimodal pain management	Yes	61 (62.2)	4	3.0-4.9	<0.001^#^
	No	37 (37.8)	5	4.0-6.0	
Proper urinary catheter placement	Yes	75 (76.5)	4	3.5-4.9	<0.001^#^
	No	23 (23.5)	5.7	4.8-7.0	
Avoiding gastric tube	Yes	96 (98)	4.1	3.7-5.2	0.209^#^
	No	2 (2)	6	4.8-7.1	
Minimally invasive surgery/limited muscle cutting	Yes	75 (76.5)	4	3.5-5.0	<0.001^#^
	No	23 (23.5)	5.9	4.8-7.0	
Patient's body temperature management	Yes	97 (99)	4.2	3.7-5.2	0.737^#^
	No	1 (1)	4	4.0-4.0	
Nausea and vomiting prevention	Yes	98 (100)	4.1	3.7-5.2	-
	No	0 (0)	-	-	
Optimal fluid management	Yes	95 (96.9)	4.1	3.7-5.2	0.046^#^
	No	3 (3.1)	7	5.0-7.9	
Use of short-acting muscle relaxants	Yes	98 (100)	4.1	3.7-5.2	-
	No	0 (0)	-	-	
Postoperative phase					
Prevention of atrial fibrillation	Yes	96 (98.0)	4.1	3.7-5.2	0.041^#^
	No	2 (2.0)	7.6	7.1-8.2	
Early postoperative physical therapy at the recovery room	Yes	93 (94.9)	4.1	3.7-5.1	0.004^#^
	No	5 (5.1)	6.8	6.1-9.9	
Early postsurgery feeding	Yes	96 (98.0)	4.1	3.7-5.2	0.097^#^
	No	2 (2.0)	6.8	5.7-7.8	
Pain evaluation and multimodal postoperative care	Yes	61 (62.2)	4	3.0-4.9	<0.001^#^
	No	37 (37.8)	5	4.0-6.0	
Timely drain removal	Yes	79 (80.6)	4	3.2-4.8	<0.001^#^
	No	19 (19.4)	7	5.9-9.3	
Continuing physical therapy in the surgical ward	Yes	97 (99)	4.1	3.7-5.2	0.223^#^
	No	1 (1)	6.8	6.8-6.8	

Table 5 shows the relationship between PLOS and the number of adherence factors. There was a negative correlation between the number of ERAS adherence factors and PLOS. Specifically, a strong negative correlation existed between the number of ERAS adherence factors during/after surgery and the PLOS (p<0.001). A moderate negative correlation was observed between the number of ERAS adherence factors before surgery and PLOS (p<0.001). Overall, an increase in adherence to ERAS factors was associated with a reduction in PLOS (p<0.001).

**Table 5:** The relationship between postoperative length of hospital stay and number of adherence factors (n=98) R: Spearman correlation coefficient #: Spearman regression

Number of adherence factors and postoperative length of hospital stay	Length of postoperative hospital stay (day)	
	Correlation coefficient	p-value ^#^
Number of preoperative adherence factors	R=-0.42	<0.001
Number of intraoperative adherence factors	R=-0.62	<0.001
Number of postoperative adherence factors	R=-0.67	<0.001
Total number of adherence factors	R=-0.74	<0.001

## Discussion

This is a single-center observational study investigating the PLOS and related factors in the implementation of the ERAS Program protocol for 98 patients undergoing lobectomy for cancer treatment. The study emphasizes the correlation between adherence to the intervention protocol and the PLOS. This study observed the median PLOS following ERAS intervention was 4.1 days, with an interquartile range of 3.7 to 5.2 days. Studies reporting evaluation of PLOS after ERAS intervention in patients undergoing lobectomy also range from three to five days [10]. This review compared the median PLOS between the ERAS and non-ERAS groups. The reductions ranged from one to three days. A statistically significant difference in PLOS between the comprehensive ERAS group and the non-ERAS group was reported in all studies. In the ERAS group, the median PLOS ranged from three to five days. In the non-ERAS group, the median PLOS ranged from four to seven days [10]. Our study also showed improvement compared to our center's previous evaluation report of PLOS before ERAS intervention. The median PLOS in preERAS assessment was 5.2 days (interquartile range 4.8 to 6.8 days) [4].

Our study results show that the majority of patients' background characteristics do not have a statistically significant relationship with the PLOS. The only factor we found an association with was smoking status. Similar studies have found an association between cigarette smoking and length of hospital stay. A study in Iran by Sari et al. [14] noted that compared to never smokers, current and former smokers had PLOS 72% and 31% higher, respectively. The role of smoking cessation has been widely recommended through previous consensus on ERAS intervention, which is that patients are recommended to stop smoking at least four weeks before surgery [5,7,9].

Regarding the relationship between PLOS and treatment characteristics, VATS had a shorter hospital stay than open thoracotomy [15]. In our study, longer hospital stays may be related to open surgery with a hospital stay of 5.4 days, 0.2 days higher than VATS converting to open surgery and 1.4 days higher than VATS. Indeed, open surgery was associated with a 10-fold increased risk for postoperative complications versus VATS and was an independent risk factor for surgical complications [16]. That is the reason why PLOS in patients with open surgery would be higher than VATS. In addition, we found that there is a strong positive correlation between the duration of thoracic drainage and PLOS (p<0.001), a moderate positive correlation between operation time and PLOS (p<0.001), and a weak positive correlation between time in recovery unit and duration of urinary catheterization (p<0.05). This also explains the prolonged hospital stay mentioned by Galata and colleagues in their study [16]. Forster et al. reported that early removal of thoracic drainage, use of electronic drainage, opioid cessation, and early oral feeding were associated with reduced rates of complications [17]. Shorter PLOS was correlated with early removal of thoracic drainage and opioid cessation.

During the ERAS intervention process, one of the very important factors that many studies have mentioned is adherence to the ERAS program steps. The adherence to ERAS protocols has been very difficult and challenging as it has depended on many aspects, including support and commitment from leadership, individual awareness of patients, knowledge, attitudes, and practice of medical staff, and good coordination of related specialties. The effectiveness of the ERAS program has been evaluated through PLOS, which has correlated with the level of adherence to the protocol. A study involving 422 patients by Rogers and colleagues has confirmed a significant inverse correlation between adherence to the protocol and the rate of postoperative complications [18]. Another study by Forster et al. involving a cohort of 192 ERAS patients undergoing pulmonary lobectomy has revealed that high adherence to the ERAS protocol, with rates ≥75%, was associated with a reduction in postoperative complication rates and a decreased incidence of delayed discharge [17]. The study findings by Madani et al. also noted that the entire ERAS process could be more significant than individual factors. However, it has been demonstrated that early thoracic drainage and urinary catheter removal are independent predictive factors for shorter hospital stays [19]. The study by Rasilainen and colleagues showed a mean ERAS adherence rate of 67%. Adherence above 70% has been significantly associated with lower complication rates and shorter hospital stays and was set as a threshold for more detailed analyses [20].

Because this study is only a single-center study and the observational design includes potential biases, the generalizability of our results to other settings with different patient populations, hospital resources, and cultural practices may be limited. In addition, the study's observational nature means causality between ERAS protocol adherence and outcomes cannot be definitively established. Future study designs could benefit from multi-center trials to validate our findings with larger populations and explore the impact of specific ERAS components in more detail.

This study has several strengths, including its prospective design and the comprehensive implementation of the ERAS protocol across various phases of patient care. Our findings are reinforced by the adherence to ERAS protocols, demonstrating a clear correlation with reduced LOS, which aligns with global research trends.

## Conclusions

Implementing the ERAS program in hospitals is feasible and effective with adequate resources and staff readiness. The success of the program depends on effective interdisciplinary collaboration and strict adherence to established protocols. By focusing on optimizing patient outcomes through a multidisciplinary approach, ERAS programs can significantly improve postoperative recovery times, reduce PLOS, and enhance the overall patient experience.
